# Barriers to Dairy Product Consumption: The Role of Information and Promotional Activities from the Perspective of Polish Physicians

**DOI:** 10.3390/foods15193374

**Published:** 2026-09-22

**Authors:** Agnieszka Komor, Aneta Jarosz-Angowska, Anna Goliszek, Anna Nowak, Artur Krukowski, Sebastian Białoskurski, Bartosz G. Sołowiej, Katarzyna E. Przybyłowicz, Katarzyna Staniewska, Aneta Dąbrowska

**Affiliations:** 1 Department of Management and Marketing, Faculty of Agrobioengineering, University of Life Sciences in Lublin, Akademicka 13, 20-950 Lublin, Poland; agnieszka.komor@up.edu.pl (A.K.); anna.goliszek@up.edu.pl (A.G.); sebastian.bialoskurski@up.edu.pl (S.B.); 2 Department of Economics and Agribusiness, Faculty of Agrobioengineering, University of Life Sciences in Lublin, Akademicka 13, 20-950 Lublin, Poland; aneta.angowska@up.edu.pl (A.J.-A.); anna.nowak@up.edu.pl (A.N.); 3 Department of Dairy Technology and Functional Foods, Faculty of Food Sciences and Biotechnology, University of Life Sciences in Lublin, Akademicka 13, 20-950 Lublin, Poland; bartosz.solowiej@up.edu.pl; 4 Department of Human Nutrition, Faculty of Food Sciences, University of Warmia and Mazury in Olsztyn, Słoneczna 45f, 10-718 Olsztyn, Poland; katarzyna.przybylowicz@uwm.edu.pl; 5 Department of Commodity Science and Food Analysis, Faculty of Food Science, University of Warmia and Mazury, Pl. Cieszyński 1, 10-726 Olsztyn, Poland; kasta@uwm.edu.pl; 6 Department of Dairy Science and Quality Management, Faculty of Food Science, University of Warmia and Mazury in Olsztyn, Oczapowskiego 7/8, 10-719 Olsztyn, Poland; anetazj@uwm.edu.pl

**Keywords:** dairy products, demand barriers, sources of information, promotional and educational activities, factor analysis

## Abstract

This study aimed to identify and evaluate physicians‘ perceptions of the key barriers that limit the consumption of dairy products, as well as the information sources and measures that could be implemented in marketing and educational practices to increase dairy product consumption. The study is based on a survey conducted among Polish doctors. Data were analysed using measures of central tendency and variability, rank analysis, and the principal component method. According to the surveyed physicians, the most important barriers were lack of proper eating habits and insufficient knowledge of the health benefits of dairy products, followed by concerns related to food adulteration and information about potential health risks for certain consumer groups. Based on principal component analysis of the surveyed physicians’ assessments, the perceived barriers to dairy product consumption were grouped into three dimensions: educational and cultural barriers, ethical and environmental barriers, and pragmatic and health-related barriers. According to respondents, doctors and recommendations from family members and friends are important sources of information about dairy products. In the physicians’ view, digital communication channels are perceived to have a greater influence on consumer attitudes and behaviours than traditional media. The surveyed doctors suggested several measures that could increase consumer interest in dairy products, including disseminating expert-endorsed messages, running social campaigns to raise awareness among children and parents, publicising recommendations issued by scientific institutions on the benefits of consuming dairy products and educating consumers about quality certification labels. This study addresses a research gap by providing an integrated assessment of dairy consumption barriers, information sources, and promotional and educational activities from the perspective of Polish physicians, thereby complementing previous consumer-focused research with a professional medical perspective. The study’s results also enable the identification of measures to increase demand for dairy products by leveraging doctors’ influence on consumer opinions in the marketing communication process.

## 1. Introduction

Dairy products play a fundamental role in the human diet, providing an important source of high-quality protein, calcium, vitamins, and bioactive compounds essential for health and normal physiological functioning [1,2]. Globally, demand for dairy products has been increasing, particularly in developing countries, driven by rising income levels, urbanisation and demographic changes [3,4,5]. In contrast, many developed countries have experienced a decline in liquid milk consumption, which is partially compensated by growing demand for yoghurt, cheese, and fermented dairy products [6,7]. Contemporary consumption trends indicate that food choices are increasingly shaped by heightened awareness of health, environmental sustainability, and animal welfare. As a result, consumers are seeking natural and environmentally friendly products, while simultaneously showing growing interest in plant-based milk alternatives to traditional milk [8]. These trends contribute to the emergence of various barriers to dairy consumption, which may stem from perceived health risks, ethical concerns, environmental considerations, or insufficient or inconsistent information available to consumers.

Understanding consumer decision-making processes requires a thorough examination of the sources of information upon which purchasing decisions are based [9]. Without identifying these sources, it is difficult to explain consumer motivations or to design effective strategies aimed at increasing demand for specific food products. The literature highlights the existence of a complex and multi-layered system of information sources, ranging from product packaging and on-pack messages [10], including traditional and digital media, mobile applications, interpersonal recommendations, and public institutions [11,12]. A comprehensive overview of food-related information sources and communication channels is provided by Meijer et al. [13]. However, empirical studies focusing specifically on dairy products remain limited. Moreover, there is a noticeable lack of research that simultaneously examines demand barriers and the relative importance of information sources influencing consumer perceptions of dairy products. This gap is particularly evident with regard to the role of medical professionals as trusted sources of information in shaping consumer attitudes towards dairy products.

Admittedly, a number of academic studies have examined the significance and influence of the Polish medical community on education in the broadest sense, as well as on the promotion of health-related issues, including healthy eating. According to Bator [14], nutrition is the most important factor in shaping human health, and its importance increases during the treatment process when a person is ill. Public health education, primarily in the areas of nutrition and physical activity, often plays a significant role in disease prevention. This process often relies on qualified medical staff. Medical staff should possess a high level of knowledge regarding healthy eating habits in particular, as health promotion should be regarded as a fundamental task alongside clinical care for all healthcare organisations. Providing health education for patients can be highly beneficial, both in terms of healthcare and for society as a whole. The author’s research shows that doctors and nurses have a statistically significantly greater level of nutritional knowledge than other healthcare workers who took part in the study. The author argues that general practitioners (GPs) should play a key role in educating patients. It transpires that 90 per cent of patients’ interactions with the healthcare system consist of visits to their GP. Furthermore, the results of other studies [15] indicate that around 60 per cent of primary care doctors reported providing health education to their patients. Furthermore, doctors are generally regarded as the most competent source of information regarding a patient’s state of health or a specific illness. Their personal example, i.e., their lifestyle and awareness of their status as role models, is a key driver of health-promoting activities. Doctors’ lifestyles influence not only doctor–patient consultations and patient care, but also their own health. It should be noted that the health of medical staff is a priority for the public health system [16].

Research by Znyk, Zajdel and Kaleta [17] indicates that GPs influence patients’ health behaviours. One in three patients (36%) consulted their GP about their diet and eating habits, while 39% consulted them about physical activity. One in three people received advice on nutrition and physical activity. Such discussions were more common among obese individuals (60%) than among those who were overweight (39%). Interestingly, the initiative to discuss nutrition or physical activity mainly came from the doctor (71%) rather than the patient. Meanwhile, patients perceive doctors as the most reliable source of knowledge on nutrition [16].

In light of the above considerations, the article aimed to identify and evaluate physicians’ perceptions of the key barriers to dairy product consumption, as well as the sources of knowledge and activities that could be employed in marketing and educational practices to boost consumption of dairy products. This was based on a survey conducted among a sample of Polish doctors. This is a significant area of research and practical importance. The following research questions were formulated to achieve the main objective: RQ1: What factors, according to responding doctors, negatively impact the level of dairy product consumption? RQ2: Based on the respondents’ opinions, what groups of barriers (principal components) limiting the consumption of dairy products can be identified? RQ3: How do responding doctors assess the perceived influence of individual sources of information on consumer opinions and attitudes towards dairy products? RQ4: What importance do responding doctors attach to information and promotional activities in shaping dairy-product consumption?

Doctors were chosen as a group of respondents because of their unique position within the communication system concerning health and nutrition. As medical professionals, they have specialist knowledge of the health determinants of dietary patterns. Thanks to their direct contact with patients, they can also observe recurring questions, beliefs, concerns and problems relating to dietary choices. From the perspective of the Theory of Planned Behaviour (TPB), doctors can also be considered a key element in shaping subjective norms and beliefs about the health consequences of specific dietary behaviours. Their significance is further justified by the high level of trust placed in professional health information sources and the potential influence of medical recommendations on patients’ dietary decisions. For this reason, doctors constitute a suitable group for assessing the perceived importance of barriers to dairy product consumption, sources of information, and educational and promotional activities related to their consumption. However, it should be emphasised that the results reflect doctors’ professional opinions and assessments of factors that may influence consumer behaviour rather than direct measurements of consumers’ behaviour, attitudes or decision-making processes. Therefore, they should be interpreted as an expert perspective rather than as a basis for generalising conclusions to the entire consumer population.

The contribution of this study lies in integrating three dimensions that have largely been examined separately in previous research: perceived barriers to dairy consumption, the relative importance of information sources, and the potential usefulness of educational and promotional activities, all considered from the professional perspective of Polish physicians.

## 2. Literature Review

### 2.1. Theoretical Foundations of the Analysis of Barriers to Purchasing Dairy Products

Models of consumer behaviour are widely used theoretical frameworks that help to explain consumers’ decision-making processes when purchasing goods and services. A literature review by Naskręt and Pierański [18] shows that the Theory of Planned Behaviour (TPB), proposed by Ajzen [19], is the most frequently used theory for analysing consumer behaviour in the food market. Similar conclusions were reached by Hojjati et al. [20], who emphasised the merits of applying this theory to food products. They mention the following merits: a focus on psychological factors influencing consumer behaviour; flexibility; broad applicability across contexts; and compatibility with other theories when designing complex models. Given that consumer behaviour changes over time [21], it is important to continually update information about both purchase motives and purchase barriers in the food market. According to TPB, purchase intention results from three groups of determinants: (1) attitudes towards the product (e.g., healthiness, taste, and safety); (2) subjective norms (e.g., expectations of family or professional environments, such as physicians); and (3) perceived behavioural control (e.g., ease of purchase, availability, and price) [22,23]. The usefulness of this model in the context of dietary choices has been confirmed by numerous studies that demonstrate significant associations among attitude, subjective norms, perceived behavioural control, intentions, and actual dietary behaviours [24,25,26]. In relation to dairy products, this means that consumers may limit their purchases due to personal beliefs about the products’ health and value, the influence of their social environment, and the perceived difficulties of purchasing or consuming them. The TPB model can also be extended or modified by including more predictors of intentions and behaviours [27]. Hansen et al. [28] refer to the theory of expected value in their research, according to which consumers’ purchasing decisions are based on their assessment of the benefits, success and value of a given purchase. The consumer’s assumed goal is to maximise consumption benefits. This approach forms an important basis for Ajzen’s Theory of Planned Behaviour. In TPB, attitude towards behaviour stems from beliefs about the consequences of the behaviour and how those consequences are evaluated.

Demand for dairy products is shaped by a complex combination of factors that influence consumption either positively or negatively [29,30]. Economic factors such as income and price levels, as well as the availability of substitutes, play a significant role. Demographic, cultural and health factors are also important, including growing consumer awareness, lifestyle changes and evolving food preferences. Understanding the impact of these factors is essential for identifying barriers to dairy product consumption, which is key to analysing consumer behaviour and the challenges facing producers and distributors. This knowledge is also essential for formulating effective market strategies and food policy. Although there is literature on individual factors that limit demand for dairy products, there is a lack of studies that comprehensively analyse the significance of such barriers.

The literature emphasises various barriers to consuming milk and other dairy products. Studies by Morelli and Vitale [31] and Allen et al. [32] suggest that health concerns, such as the desire to reduce fat, cholesterol and lactose intake, are among the most important reasons for limiting dairy product consumption. Digestive problems related to certain products are also cited as a significant reason for reducing dairy product consumption, especially cheese [33]. The literature also mentions ecological arguments and animal welfare issues as barriers to milk and dairy product consumption. These issues are increasingly being highlighted as reasons for avoiding milk [34,35,36,37]. One study examined the extent to which media campaigns encouraging consumers to replace dairy products with plant-based alternatives are persuasive, and thus whether they may serve as a potential barrier to dairy product consumption. They demonstrated that ethical (animal welfare) and ecological (environmental impact) factors affected consumers in the countries studied (the United Kingdom, Germany and Poland) differently. The studies also showed that, in all countries examined, dairy products were perceived more positively than plant-based alternatives in terms of most analysed attributes. Bracey et al. [38] investigated the relationship between dairy product consumption and animal welfare and ecological concerns. Their research showed that consumers with high dairy intake had a more positive attitude towards dairy cattle farmers than consumers with lower intake. Conversely, low intake was associated with greater concern for animal welfare and the environmental impact of dairy product production.

According to the literature, economic factors, including the prices of dairy products, limit their consumption [6,39,40]. However, the magnitude of this effect varies depending on the product type, market conditions and the socio-demographic characteristics of respondents, as well as the region under consideration [41,42]. Gehrke and Jin’s findings [43], based on a study conducted across 27 EU countries, indicate that milk and dairy products are relatively sensitive to price changes, with lower-income households exhibiting greater price sensitivity. Irz and Kuosmanen [44] reached slightly different conclusions for Finland, finding that the low price elasticity of demand for dairy products limits the impact of price changes on consumption. Another factor limiting dairy consumption is concern about the safety of these products, particularly in relation to food fraud and scandals. For instance, a survey conducted one year after the melamine scandal in China was disclosed showed that some households had substantially reduced their liquid milk consumption, while others had eliminated milk from their diets entirely [45]. Respondents identified the loss of confidence in the safety of dairy products as the direct reason for reducing their consumption. Research also indicates that, once lost, trust is very difficult to restore. Indeed, the effects of the melamine scandal in China continued to significantly undermine trust in actors across the dairy supply chain for many years afterward [46].

The literature also identifies factors that limit dairy consumption, including poor dietary habits and low nutritional knowledge. Wei and Wang [47] confirmed that insufficient knowledge about dairy products is a significant issue among populations with inadequate dairy consumption. Research conducted among children in Poland showed that higher levels of nutritional knowledge among children and their parents were associated with more frequent consumption of dairy products. Family dietary habits also play an important role in milk and dairy product consumption, particularly the practice of eating meals together [30]. As dairy products form part of broader dietary behaviour patterns, their low consumption may be both a consequence of inadequate dietary habits and an indicator of an overall unhealthy dietary pattern. Furthermore, the literature indicates that dietary trends promoting plant-based foods, including milk alternatives, may limit dairy consumption [48]. Research by Capps and Wang [49] demonstrates that plant-based beverages act as substitutes for conventional and organic milk. This suggests that increased demand for plant-based alternatives may reduce demand for conventional milk. Similar findings, particularly regarding soy-based products, were reported by Yang et al. [50].

### 2.2. Information and Promotion Activities on the Dairy Products Market

Promotion should be considered as part of the wider marketing communications concept, which focuses on building relationships with various market participants, including final consumers [51,52]. A company’s target audience includes not only potential buyers, but also a wide range of recipients of informational and promotional content, including internal and external stakeholders [53,54,55].

Using promotional activities in different markets is important for conveying information about specific products. The choice of tools and how intensely they are used depends on many factors (e.g., product nature, market size and buyer characteristics). In the case of dairy products, for example, consumption is generally widespread and mass-market, meaning that promotional instruments must take this into account.

In the dairy products market, advertising in the mass media (television, radio and billboards) remains the basic form of marketing communication, and sales promotion is becoming increasingly important. Advertising messages often emphasise the importance of meeting the body’s calcium and protein requirements for consumer health [56]. Advertisements using emotional messages and health claims are effective in convincing parents to choose dairy products. These messages often emphasise the nutritional benefits and convenience of consuming dairy products, making them attractive to health-conscious consumers [57].

Consumer decisions in the dairy market are increasingly influenced by current sales conditions. This is the result of dynamic changes in manufacturers’ offerings, combined with high product availability thanks to new trade organisations. This is largely generated by sales promotion activities. The influence of this form of promotion on the purchasing behaviour of store customers within new trade organisation solutions is a dynamic process that competes with the impact of basic market communication tools [58].

Due to socio-cultural changes among buyers, especially young people, digital promotional tools such as social media and influencer marketing are becoming increasingly important in the dairy products market. According to research by Ng et al. [59], using influencers on social media to promote dairy products can significantly boost engagement and reach. Influencer partnerships play a significant role in promoting dairy products by leveraging the strong bond between influencers and their followers to increase brand visibility, shape consumer perceptions and boost purchase intent [60].

In addition, tools such as branded websites, social media and mobile apps can attract and retain customers by creating engaging content. These platforms offer additional opportunities for contact and relationship building, enabling consumers to interact with the brand and find out more about its products [3,61].

When analysing information and promotional activities as tools for increasing demand for dairy products, it is important to consider the concept of socially responsible marketing used by dairy producers. According to Popowska and Starnawska [62] and Vasilev [63], it encompasses a variety of market activities undertaken to solve social problems. This includes educational promotional campaigns, for example. There has been a general increase in interest in social marketing for some time now, and it is becoming more prevalent in health prevention activities. It is being used increasingly to promote a healthy lifestyle in Poland and other countries [64]. The results of the research indicate that such social campaigns (e.g., the ‘Drink milk, you’ll be great’ campaign or the ‘Drinking pure fat’ campaign broadcast in the USA) provide information on the health benefits of consuming dairy products. It should be emphasised that public health promotion activities in most developed countries encompass appropriate health education, environmental modifications and the cultivation of healthy dietary habits. These activities empower individuals to take control of their own health and well-being. Lifestyle-oriented educational programmes have been shown to positively influence behavioural changes, particularly with regard to diet and physical activity [65,66,67]. Numerous public institutions in Poland and worldwide organise and fund social campaigns that promote the consumption of a diverse range of food products, including dairy, with the aim of improving consumer health and well-being. Furthermore, the content of these campaigns often dispels dangerous myths surrounding food products. These myths often relate to health issues concerning cardiovascular disease and metabolic disorders, as well as the composition, properties and manufacturing technologies of food products. Many of these beliefs lack scientific substantiation or oversimplify complex scientific evidence, constituting misinformation that can significantly and adversely influence consumer purchasing decisions [68,69,70].

## 3. Materials and Methods

To achieve the aim of this study, an empirical survey was conducted in Poland. Primary data were collected using a questionnaire survey. A total of 201 (N = 201) interviews were conducted using the CAPI (Computer-Assisted Personal Interviewing) method to ensure complete responses and reduce missing data. The study was funded by the Minister of Science and Higher Education of the Republic of Poland as part of the project titled “The Research Network of Life Sciences Universities for the Development of the Polish Dairy Industry—Research Project.” The project has various objectives, including promoting a sustainable approach to public health by encouraging dairy consumption and developing an innovative model to disseminate new knowledge about its health-promoting properties. The project also aims to improve the safety, quality and health-promoting attributes of dairy products, and to support pro-environmental initiatives within Polish dairy processing plants.

The questionnaire survey was conducted between 23 September 2024 and 25 April 2025. At the beginning of the survey, respondents were informed about the project under which the research was being conducted (the project details and funding sources are provided at the end of the manuscript) and that personal data were processed in accordance with the European General Data Protection Regulation (GDPR). Respondents were informed at the beginning of the questionnaire that they could withdraw from the survey at any time and the completion of the questionnaire was regarded as informed consent to participate in the research project and confirmed that they had read and understood the research procedure. The respondents were recruited from among physicians registered on dedicated online research panels maintained by the company conducting the survey, while all interviews were conducted using the CAPI method. The online panels were used solely as a source for respondent recruitment and were not used for data collection. Due to GDPR, no personally identifiable information was disclosed to the researchers during or after data collection. This study was conducted according to the guidelines of the Declaration of Helsinki, and approved by the Bioethics Committee of the Faculty of Medical Sciences, University of Warmia and Mazury in Olsztyn, Poland, on 30 March 2023 (Resolution No.11/2023).

The sample was selected using a non-probability quota sampling procedure with proportional regional allocation, incorporating elements of modified area sampling and additional controls for age and gender. Participation in the study was limited to practicing physicians who had direct contact with patients. These criteria constituted the eligibility requirements for participation in the study. The identity of the participants was verified using their medical licence numbers in official registers. As the study was conducted in all provinces of Poland, it was a nationwide study. The number of physicians included in the study in each province was determined in proportion to the number of inhabitants of that province. All provincial capitals were included in the study, and the established quotas were complemented by respondents from randomly selected smaller towns. Although individual physicians were not selected through probability sampling, the sampling procedure ensured broad geographical coverage and territorial diversity of the sample. Additionally, the distribution of respondents by age and gender was controlled to reflect the actual age and gender structure of the physician population in Poland according to the Central Statistical Office data, thereby improving the correspondence of the sample with the demographic structure of the physician population. A total of 201 Polish physicians completed the survey. The researchers did not have access to data on the total number of physicians invited to participate, refusals, or discontinued interviews. Consequently, it was not possible to reliably calculate the response rate or completion rate. The final sample included 120 women (59.7%) and 81 men (40.3%). The respondents were distributed across the following age groups: 25–34 years old (14.9%), 35–44 years old (31.3%), 45–54 years old (30.3%), and aged 55 years and over (23.4%).

The physicians surveyed represented 23 different medical specialties. The most frequently cited specialties were family medicine (n = 75), internal medicine (n = 75) and paediatrics (n = 57). Respondents could indicate more than one specialty. Specialty was not a criterion for the deliberate selection of respondents.

A research instrument in the form of a proprietary questionnaire was developed for the specific purposes of the study. The questionnaire comprised a number of questions, including demographic details and subject-specific enquiries. The survey’s scope encompassed a range of inquiries, including the perceived health benefits of consuming dairy products, medical recommendations concerning the frequency of dairy consumption, the perceived role of dairy products in the prevention and treatment of selected conditions, factors influencing purchasing decisions regarding dairy products, sources of information and knowledge about dairy products, barriers hindering dairy consumption, and potential promotional and educational measures that could enhance consumer interest in these products. The survey questionnaire was developed using the results of a cognitive-critical analysis of the literature, as well as data obtained through the systematic consensus-building method (the Delphi method—three rounds). However, it should be noted that no formal psychometric validation of the entire tool was carried out, which may be considered a limitation of the study. The present paper sets out the findings of research undertaken on three questions, which constituted merely a fraction of a more extensive questionnaire and were utilised to attain specific research objectives. The aforementioned inquiries were presented in the form of closed-ended questions in a tabular format, each of which concerned a separate area of research. The questions were as follows:

(1) To what extent, in your opinion, do the indicated factors have a negative impact on the consumption of dairy products?
(2) To what extent do you think the indicated sources of information and knowledge influence the opinions and attitudes of consumers of dairy products?
(3) To what extent could the presented measures contribute to increasing dairy product consumption among consumers?

A detailed description of these questions, together with the possible response options, is provided later in the manuscript. It is also important to emphasise that, in addition to the response options that had been previously defined, an ‘other’ option was included for each of the three questions analysed. This approach enabled respondents to identify additional factors, sources of information, or actions that were not included in the basic set of response options but that, in their view, might be relevant to the issue under analysis. Consequently, the questionnaire design did not restrict respondents’ answers to the categories identified during the survey tool’s preparation. The physicians were asked to assess barriers to dairy product consumption, as well as the sources of information and knowledge influencing the formation of opinions and attitudes of dairy product consumers in general, that is, without distinction by age, generation, or other sociodemographic characteristics. For each question, respondents rated a set of variables determined on the basis of a review of the literature on the subject using a five-point Likert ordinal scale. The use of an ordinal scale enabled a quantitative assessment of physicians’ perceptions of the importance of individual barriers to dairy consumption, information sources, and educational and promotional activities potentially influencing consumer attitudes towards dairy products. The respondents’ assessments were subsequently analysed using descriptive statistical methods. Given the ordinal nature of the five-point Likert scale, the median (Me) was calculated, while the arithmetic mean (M) was used as a supplementary descriptive measure to facilitate comparison of the relative levels of ratings across individual variables. The variability of responses was characterised using the standard deviation (SD), which was also treated as a supplementary descriptive measure. Additionally, a ranking analysis was performed, assigning ranking variables to individual variables based on their mean ratings. This allowed the identification of consumption barriers, sources of information and activities that were perceived as the most and least important in shaping consumer opinions and attitudes towards dairy products. To deepen the analysis of the structure of the variables that determine barriers to the consumption of dairy products, the principal component method was also applied (PCA) as an exploratory data-reduction technique. This procedure aimed to reduce the number of analysed variables by identifying principal components that group similar barriers. The data were standardised prior to analysis. Before proceeding with the actual analysis, the reliability of the measurement scale was checked using Cronbach’s alpha coefficient, which made it possible to assess the internal consistency of the questionnaire items. The analysed catalogue of eight variables related to purchasing barriers achieved an alpha coefficient value of 0.787 for all respondents, indicating acceptable reliability of the scale in the social sciences and proving its internal consistency. Preliminary tests were also conducted to evaluate the suitability of the data: Bartlett’s test of sphericity and the Kaiser–Meyer–Olkin (KMO) measure of sampling adequacy. A KMO value above 0.7 and the significance of Bartlett’s test at *p* < 0.001 confirmed the suitability of the data for PCA [71]. The number of principal components retained was determined based on a comprehensive assessment of several criteria. The shape of the Cattell scree plot, the proportion of variance explained by successive components, and the substantive interpretability of the solution obtained were all taken into account in the analysis. The Kaiser criterion (an eigenvalue greater than 1) was not treated as a decisive criterion. A limitation of the procedure adopted for determining the number of principal components is the omission of parallel analysis as an additional retention criterion. Next, using Equamax rotation with Kaiser normalisation was then applied to obtain a clearer structure of loadings and assign individual variables to the extracted principal components. Within each component, the variables with the highest loadings relative to a given coefficient were identified (i.e., variables with values of at least 0.5, the limit commonly adopted in the literature). On this basis, variables were assigned to the identified components. The reliability of the sets of items assigned to the individual components was assessed using Cronbach’s alpha coefficients and inter-item correlations, the latter serving as a complementary measure of internal consistency for components comprising two or three items [71]. The validity of the interpretation of the extracted structure was assessed on the basis of the magnitude of the component loadings, the conceptual coherence of the items assigned to the individual components, and their consistency with the adopted theoretical assumptions. Statistical analysis of the collected primary data was performed using PS IMAGO PRO 10.0.

A limitation of this study is that no separate pilot study was conducted prior to the main study. This was primarily attributable to the particular composition of the study group, which consisted of doctors in direct contact with patients. Due to the nature of their profession, it is challenging to recruit these respondents to participate in questionnaire-based surveys. Conducting an additional pilot phase involving respondents meeting the established criteria could have further limited the ability to achieve an adequate sample size in the main study and increased the time burden on respondents. Concomitantly, given the professional nature of the study group and the scope of the issues addressed, it was assumed that the questions and terminology used would be comprehensible to the respondents. Nevertheless, the absence of a pilot study precludes the opportunity for an early empirical evaluation of the complexity and clarity of individual questionnaire items.

## 4. Research Results and Discussion

### 4.1. Factors Negatively Affecting the Consumption of Dairy Products in the Opinion of Doctors

To explore medical professionals’ views on factors that negatively affect consumer dairy consumption, respondents were asked to rate eight potential barriers on a five-point scale. The barriers were identified based on the literature of the subject [38,48,72,73,74,75,76,77,78]. Of these barriers, two were related to health issues (insufficient knowledge of health benefits and information indicating potential health risks for certain groups, e.g., those with food allergies). In the TPB model, these barriers are primarily linked to behavioural beliefs, which form the basis for shaping attitudes towards specific behaviours. If consumers lack sufficient knowledge of the potential benefits of consuming dairy products, they may value their consumption less and consequently display a less positive attitude towards purchasing and consuming them. However, information about potential health risks may lead to negative beliefs about the consequences of consumption, even if these risks only affect certain consumer groups. Such mechanisms have been observed in studies on lactose-free products. Yılmaz et al. [79] demonstrated that knowledge of and information about intolerance and health influence consumers’ purchasing behaviour, and that insufficient information was one of the factors limiting the purchase of lactose-free products.

Three barriers were related to the image of the milk production and processing sectors (perceived negative environmental impact of dairy farming, ethical issues related to industrial livestock farming and animal welfare, and food scandals, e.g., food adulteration). These factors can be classified as sources of behavioural beliefs as they relate to the anticipated consequences of choosing and consuming a particular product. A study of dairy product consumers in the United Kingdom found that those with lower consumption levels were more concerned about animal welfare and the environmental consequences of milk production. In contrast, higher consumption was associated with a more positive attitude towards the dairy sector [38].

Two barriers were related to consumer preferences (poor eating habits and food trends favouring plant-based products, e.g., soy-based beverages). The identified barriers can be interpreted in light of the theory of planned behaviour (TPB), which states that an individual’s behaviour is preceded by their intention to perform it. This intention is shaped by three fundamental elements: attitude towards the behaviour, subjective norms and perceived behavioural control.

The final barrier was related to market issues (high product prices) (Table 1). This barrier should be considered as an additional factor that may influence perceived behavioural control or the transition from intention to action. In the context of the TPB model, Ajzen [22] explains that perceived behavioural control refers to an individual’s belief in the likelihood that a given behaviour will occur. This encompasses elements related to available resources and capabilities, among other things. Therefore, price can be interpreted as an external factor that limits the possibility of purchase and thus influences perceived control.

Respondents were asked to evaluate the discussed barriers using their professional knowledge, experience and general opinion of the factors that influence the consumption of dairy products among consumers. The instructions did not require responses to refer exclusively to direct observations of specific patients’ behaviours or any particular consumer group. The responses obtained should be interpreted as physicians’ opinions on potential determinants of consumption, rather than as direct measures of actual consumer behaviour or individual motivations.

First of all, RQ1 was addressed. The mean ratings of the above barriers range from 3.48 to 4.07. According to the responding doctors, the main barrier to dairy consumption is a lack of proper eating habits (M = 4.07; Me = 4.0), indicating that it is considered to be important by a large majority of respondents. This barrier was assessed as large or very large by 75.6% of respondents, further confirming its importance as a limiting factor in dairy product consumption. The second-highest rated barrier was insufficient knowledge of the health benefits of dairy products (M = 3.96; Me = 4.0), highlighting the importance of education in shaping consumer attitudes. The significance of these barriers has also been confirmed in other studies. For example, Myers et al. [80] demonstrated that educating people who consumed insufficient amounts of dairy about the nutritional value of dairy products increased their knowledge, as well as their purchases and consumption of such products. Furthermore, a study conducted among children showed that consuming the recommended amount of dairy products was associated with healthier dietary habits and behaviours [81].

The third-highest rated barrier was food scandals, e.g., those related to food adulteration (M = 3.88; Me = 4.0), which raise concerns about the sector’s credibility and food safety. Fourth place among the barriers to consumption in the opinion of responding physicians was information indicating the potential health risks of dairy products for certain consumer groups (e.g., those with food allergies), with an average rating of 3.84 and a median rating of 4. More than 70% of respondents considered the impact of each barrier in this group to be significant or very significant, indicating that these factors were considered important in limiting the consumption of dairy products. According to the surveyed physicians, negative publicity surrounding food adulteration and information indicating potential health risks for certain consumer groups, such as those with food allergies, were also perceived as important factors potentially inhibiting the consumption of dairy products, in addition to the lack of proper eating habits established early in life and insufficient education about the health benefits of dairy products.

Research findings suggest that safety risks and fraud relating to dairy products, as well as widely publicised food scandals and health concerns, may limit consumption by increasing the perceived risk and causing a loss of consumer confidence. Maitiniyazi and Canavari [82] demonstrated that the prevalence of food safety incidents causes consumers to lose confidence in food safety and pay closer attention to media reports of such incidents, including on social media. Factors contributing to a reduction in dairy consumption include health-related issues such as lactose intolerance and cow’s milk allergy, which are becoming more prevalent globally [48].

Respondents rated dietary trends related to plant-based food consumption as moderately important (M = 3.74; Me = 4.0). In this case, 64.7% of surveyed physicians considered this factor to have a significant impact on reducing dairy product consumption. This is confirmed by research by Wolf et al. [83], which showed that cow’s milk consumption in the US has declined, driven by rising consumption of plant-based drinks. Nevertheless, total dairy consumption in the United States continues to increase due to higher consumption of other dairy products, such as cheese, butter and yoghurt. An analysis of the purchasing behaviour of young consumers in Poland confirmed a link between self-reported dietary patterns and the frequency with which plant-based drinks were consumed as an alternative to traditional milk [84]. Consequently, a significantly higher frequency of plant-based drink consumption was found among those following a vegan diet. This clearly indicates that dietary trends related to the consumption of plant-based foods may be a more significant factor in limiting dairy consumption for certain consumer groups and certain types of dairy products.

Lower average values were assigned to ethical issues concerning intensive livestock farming and animal welfare (M = 3.59, Me = 4.0), high product prices (M = 3.53, Me = 4.0), and the negative perception of the dairy industry’s environmental impact (M = 3.48, Me = 4.0). On average, 55% of respondents perceived these factors as having a strong or very high influence on limiting dairy product consumption. The mean rating for these factors was approximately 3.5, indicating that, in the opinion of the surveyed physicians, they exert a relatively moderate but noticeable influence on consumers’ dairy consumption decisions.

The research findings presented here are broadly consistent with the results of international studies, which indicate that dairy consumption depends on a number of interconnected factors. These include nutritional knowledge, perceived health benefits and risks, allergies and intolerances, dietary preferences, the availability of plant-based alternatives, and ethical and environmental considerations. Although this study was conducted on a relatively small sample of Polish doctors, the identified barriers may reflect universal mechanisms, while the relative importance of demand-side barriers may depend on cultural, institutional, economic, and market conditions. This interpretation is supported by recent comparative studies, which reveal common patterns as well as country-specific ones in attitudes towards dairy products and plant-based alternatives. For instance, Maison et al. [37] identified shared obstacles to the uptake of plant-based milk alternatives in Poland, Germany, and the United Kingdom, as well as notable variations in motivations for reducing dairy consumption across these countries. Similarly, Adamczyk et al. [85] demonstrated that the role of dairy products and attitudes towards their plant-based alternatives varied across Poland, Germany, and France in relation to culinary traditions. Consequently, this study may serve as a useful conceptual and exploratory reference for research conducted in countries with comparable socioeconomic, cultural, dietary and healthcare contexts. However, generalising these findings to different national contexts requires empirical verification.

As a result of principal components analysis, three principal components were identified based on the scree plot (Figure 1), which answers the question of RQ2.

They explain over 67% of the cumulative variance (see Table 2). The KMO (Kaiser–Meyer–Olkin) index, a measure of variable selection adequacy, exceeded the recommended threshold of 0.7, indicating the validity of the principal component analysis. Bartlett’s sphericity test was statistically significant (*p* < 0.001), confirming the relationship between the variables and the validity of the analysis.

Based on physicians’ assessments of individual barriers, three principal components were identified, representing co-occurring patterns of respondents’ opinions regarding potential reasons for limiting dairy product consumption. Each component groups barriers that were evaluated similarly by physicians, while high component loadings indicate which variables contribute most strongly to a given dimension of perceived barriers. The identified dimensions do not represent consumer segments or empirically confirmed determinants of actual consumption; rather, they reflect the structure of opinions within the surveyed group of physicians (see Figure 2 and Appendix A).

The first component comprised variables related to a lack of knowledge and established eating habits. The highest component loadings were recorded for lack of proper eating habits (0.882), lack of sufficient knowledge about health benefits (0.818) and, to a lesser extent, dietary trends relating to the consumption of plant-based products (0.622). These can be described as educational and cultural barriers resulting from a lack of nutritional awareness and the growing popularity of alternative dietary models. This result indicates that physicians tend to perceive these factors as interrelated potential barriers to consumption.

The second component focuses on ethical and environmental issues. The highest loadings were found for the following variables: ethical concerns regarding industrial farming and animal welfare (0.805), and the perception of the dairy industry as having a negative environmental impact (0.764). This principal component can be interpreted as ethical and environmental barriers related to increasing consumer concern for responsible production and environmental protection issues.

The third component has a clear substantive interpretation. It includes pragmatic barriers of an economic and health nature. High component loadings were obtained for the following variables: high product prices, food ‘scandals’, and information indicating potential health risks. The loadings of these variables are 0.815, 0.702, and 0.580, respectively, further indicating a strong association between these variables and the identified component. This principal component can be described as pragmatic and health-related barriers, emphasising the importance of purchase costs and consumer concerns about food safety. As with the other factors, this result should be interpreted as the structure of physicians’ opinions regarding potential consumption constraints, rather than as direct evidence of the existence of such a pattern among consumers.

The reliability analysis revealed varying but generally acceptable levels of internal consistency among the items assigned to the extracted principal components. For the first component, Cronbach’s alpha was 0.75. Inter-item correlations ranged from r = 0.428 to r = 0.612, with a mean inter-item correlation of r = 0.503. The second component, comprising two items, yielded a Cronbach’s alpha of α = 0.620, with an inter-item correlation of r = 0.449. For the third component, Cronbach’s alpha was α = 0.653; inter-item correlations ranged from r = 0.270 to r = 0.528, with a mean inter-item correlation of r = 0.385. Overall, the results indicate acceptable internal consistency for the first component and moderate, yet acceptable in the context of exploratory analysis, internal consistency for the second and third components. The extracted structure was also substantively interpretable, and the variables assigned to each component exhibited loadings of at least 0.50, providing support for the validity of the interpretation of the identified dimensions.

In summary, principal components analysis revealed that barriers to dairy consumption fall into three distinct categories: educational and cultural; ethical and environmental; and pragmatic and health-related. These results suggest that, according to the surveyed physicians, barriers to dairy consumption are multidimensional, encompassing cognitive and cultural factors, image issues, and practical considerations related to economic and health-safety concerns.

The extracted structures describe how potential barriers are perceived by physicians rather than actual consumer segments or direct predictors of levels of dairy product consumption. Respondents’ answers may have been influenced by their knowledge, professional experience (including interactions with patients) and their own beliefs and subjective assessment of consumer behaviour. Therefore, the results should be interpreted as identifying areas that, from the perspective of the medical community, may require further research among consumers, as well as potential informational and promotional activities.

### 4.2. Physicians’ Assessment of Information Sources and Measures Potentially Contributing to Increased Interest in Dairy Products

Traditionally, food manufacturers and distributors were the main providers of food-related information [86]. However, changes in consumer preferences have increased the importance of other sources of information and communication channels. This has prompted manufacturers to adopt broader communication strategies, including digital and traditional media. This is also because consumers differ in their information needs. Sources of information and knowledge therefore play a key role in shaping consumer opinions and attitudes towards food products, including dairy products. In this context, the study aimed to determine the extent to which doctors, as health experts, perceive the influence of various information channels on consumer decisions and behaviour. The results may be of practical importance for the educational and promotional activities of dairy companies, as well as for programmes supporting healthy eating habits, as they facilitate more effective communication of consumer expectations and needs.

Doctors were asked to assess the importance of various sources of information in shaping consumers’ opinions and attitudes towards dairy products, which answers the question RQ3. The sources of consumer information presented to the surveyed doctors for assessment included social and expert recommendations (e.g., recommendations from family or friends, celebrity opinions, and recommendations from experts such as doctors and scientists); commercial sources controlled by marketers (e.g., sales recommendations and promotional activities of manufacturers such as sponsorship and participation in trade fairs); and non-commercial sources (e.g., public sources such as schools, social organisations, and social campaigns). The doctors were also asked to express their opinion on the importance of traditional marketing communication channels (radio, television and the press) compared to digital channels, including the internet and social media (e.g., Facebook, TikTok and Instagram).

An analysis of the results presented in Table 3 suggests that surveyed physicians perceived the Internet (M = 4.06; Me = 4.0) and doctors as health experts (M = 3.92; Me = 4.0) as particularly important sources potentially influencing consumer perceptions of dairy products. However, this result should be interpreted with caution. Physicians evaluated themselves as a source of information, which must be regarded as a limitation of the study. Their responses may have been biased by subjective assessments and a tendency to overestimate or underestimate their actual influence on consumers’ knowledge and dietary decisions. Social media (M = 3.87; Me = 4.0) and family recommendations (M = 3.84; Me = 4.0) were also perceived as relatively influential. The relatively low coefficients of variation indicate a high degree of consistency in respondents’ assessments. For this group of information sources, between 67% and 75% of the surveyed physicians perceived their influence on consumer attitudes and behaviours as high or very high. The group of information sources perceived by the surveyed physicians as having a moderate impact on consumer attitudes and behaviours primarily included recommendations from scientists (M = 3.75; Me = 4.0), recommendations from friends (M = 3.73; Me = 4.0), and schools (M = 3.70; Me = 4.0), as well as social campaigns promoting dairy products (M = 3.71; Me = 4.0). According to the respondents’ assessments, the least influential sources were recommendations from sales staff (M = 3.19; Me = 3.0), recommendations from social organisations (M = 3.27; Me = 3.0), and promotional activities undertaken by producers (M = 3.42; Me = 3.0).

Among the analysed sources of information and knowledge about dairy products, interpersonal sources received the highest ratings, with the surveyed physicians assigning particular importance to doctors as a source of information. This finding should be interpreted with caution, as the respondents were evaluating the potential influence of their own professional group. Furthermore, the study did not directly assess how often patients consult their doctors specifically about dairy products, nor the extent to which such consultations influence their dietary choices. Consequently, the elevated status accorded to doctors as a reliable source of information should not be construed as substantiation of the true frequency or scope of physician–patient interactions concerning dairy consumption. Previous research also indicates that doctors are among the most important sources of health information and are perceived by patients as reliable and competent. Research indicates that recommendations made by medical professionals significantly impact nutritional decisions [87]. Trust in doctors among consumers is significantly higher than trust in other sources of knowledge, such as the mass media, the Internet or advertisements, which are often perceived as less reliable and more susceptible to commercial influence [88].

Recommendations from family and friends received high ratings among the interpersonal sources of information assessed by the surveyed physicians. Specifically, 72.2% and 68.7% of respondents, respectively, perceived family and friends’ opinions about dairy products as having a strong or very high influence on consumers’ attitudes and behaviours. By contrast, celebrity recommendations were perceived by the surveyed doctors as less influential. Commercial sources, including recommendations from sellers and companies’ promotional activities, were rated lower than non-commercial sources, such as schools or social campaigns. These findings are consistent with previous research showing that interpersonal communication increases product awareness and often convinces people to try something new [89]. Earlier studies also indicate that consumers often share opinions and information about products with their friends, and that word-of-mouth marketing can influence purchasing behaviour. In addition, previous research highlights the significance of experts, including scientists, dietitians and doctors, as well as independent organisations and public institutions, in shaping consumers’ food-related purchasing attitudes. Such sources are generally considered more credible than commercial entities [90].

It is also worth noting that, according to the surveyed physicians, digital channels, particularly the Internet and social media, were perceived as having a greater influence on consumers’ attitudes and behaviours towards dairy products than traditional media such as radio, television and the press. The respondents also perceived social media as an important source potentially influencing consumer behaviour. This assessment is consistent with findings from previous studies indicating the declining relative importance of traditional information channels, particularly among younger consumers [57,91]. However, Wang et al. [92] showed that traditional media continue to shape consumer awareness, albeit to a lesser extent than digital content, particularly among younger groups. Previous research also indicates that the Internet is widely used as a source of food-related information because of its accessibility [91]. Today, social media’s role in purchasing decisions is growing, particularly among younger consumers. These sources are used for culinary inspiration, product reviews, brand marketing through celebrities and ‘micro-influencers’, and for disseminating health content [93].

On social media, users may follow, comment on and imitate the food choices of individuals they trust or perceive as credible authorities [94]. While information disseminated through multiple social media platforms can positively influence consumer behaviour [93], these platforms may also facilitate the spread of misleading and potentially harmful messages and rumours [95]. In addition, barcode scanning applications and websites that enable consumers to compare the nutritional or health-related attributes of food products are becoming increasingly popular [94].

The next step was to assess which activities, in the physicians’ view, could contribute to increasing consumer interest in dairy products. Respondents were therefore asked to evaluate the potential influence of selected marketing, educational, and promotional activities on consumer purchasing decisions, thereby addressing RQ4 (see Table 4).

Three of the assessed measures concerned awareness-raising or promotional campaigns for dairy products. Two initiatives were related to disseminating positive opinions and recommendations from experts and scientific institutes. Two others referred to the product itself (information on the label) and the image of the dairy industry. The mean ratings assigned by the surveyed physicians ranged from 3.68 to 4.08. The highest-rated initiatives, with mean scores exceeding 4.0 (Me = 4), were the dissemination of positive expert opinions, social campaigns aimed at raising awareness among children and parents, and publicising scientific institute recommendations on the benefits of consuming dairy products. More than 77% of respondents perceived these activities as potentially having a high or very high contribution to increasing consumer interest in dairy products. Awareness campaigns concerning quality certification labels displayed on packaging also received a high mean rating (M = 3.99), with 74.1% of respondents assessing their potential contribution as important or very important. The relevance of consumer education in this area is supported by previous research. Iwanicka [96] showed that consumers differ in how they perceive and interpret the quality of dairy products, while Grębowiec [97] emphasised that the large number of certification systems may limit consumers’ ability to recognise and understand them. These findings suggest that education in this area may support consumers in making more informed purchasing decisions.

The remaining measures also received relatively high mean ratings, ranging from 3.86 to 3.68. A substantial proportion of respondents perceived these measures as having high or very high potential to increase consumers’ willingness to purchase dairy products, with the corresponding shares ranging from 67.6% for creating a positive image of the dairy industry to 61.2% for campaigns promoting dairy product consumption. These findings suggest that surveyed doctors perceived all of the analyzed activities as potentially contributing to greater consumer interest in dairy products.

## 5. Conclusions

The study showed that responding physicians perceived poor eating habits and insufficient knowledge about the health benefits of dairy products as the most important factors limiting dairy product consumption. These findings point to the perceived need for more intensive nutrition education, as reliable, evidence-based communication may help reduce the spread of unfounded beliefs and support the development of healthy dietary habits and informed consumer choices. Additionally, respondents perceived negative market messages concerning food adulteration and information indicating potential health risks as significant barriers to dairy product consumption. PCA showed that the perceived barriers identified by the surveyed physicians were multidimensional and could be grouped into three categories: educational and cultural; ethical and environmental; and pragmatic and health-related.

According to the responding physicians, important sources of information and knowledge about dairy products include interpersonal sources, such as doctors and recommendations from family and friends. The surveyed physicians perceived digital communication channels, including the Internet and social media, as having a greater impact on the attitudes and behaviours of consumers of these products than traditional media such as radio, television, and the press. Furthermore, in the respondents’ assessment, several measures can contribute to increasing consumer interest in dairy products, including the dissemination of positive expert opinions, the implementation of social campaigns to raise awareness among children and parents, publicising the recommendations of scientific institutes on the benefits of consuming dairy products, and educating consumers about the quality marks placed on packaging. These findings should therefore be interpreted as physicians’ professional assessments of factors that may influence consumer perceptions and behaviour, rather than as direct evidence of consumers’ actual behaviour or of the effectiveness of the analysed communication activities.

The results obtained have significant practical implications for the educational and promotional strategies of dairy companies, as well as for programmes aimed at promoting healthy eating habits. These findings may help tailor communication to be adapted more effectively to the diverse needs and expectations of consumers. In particular, they indicated that communication activities should not be limited solely to highlighting the nutritional and health benefits of dairy products but should also address potential concerns related to food safety, animal welfare, the environmental impact of milk production, product prices, and changing dietary preferences. Comprehensive, evidence-based communication can increase consumer trust and support informed dietary choices.

This study has several limitations that should be considered when interpreting the results. First, the study included a relatively narrow group of respondents (physicians), which, on the one hand, provides an original research perspective, but on the other, limits the possibility of generalising the results to the entire population. Simultaneously, owing to similarities in food systems, food safety regulations, and conditions in the dairy products market, the results may serve as a reference point for studies conducted in other countries with similar socio-economic and cultural conditions, particularly within the European Union. However, extrapolating these findings to other national contexts requires empirical verification, as the significance of specific barriers may differ depending on cultural, economic, and market conditions. Second, the identified barriers were assessed by physicians and reflected their professional knowledge, experience, and subjective perception of factors that may influence consumer behaviour, rather than directly measured attitudes, motivations, or actual consumer purchasing and consumption behaviours. Therefore, the main components of the identified barriers should be interpreted as patterns in physicians’ perceptions of potential barriers, rather than as direct predictors of actual dairy consumption. Furthermore, physicians’ self-assessment of their role as a source of information may have been susceptible to self-perception bias. Moreover, the study did not directly assess the frequency with which patients consult their doctors specifically about dairy consumption, nor the extent to which such consultations influence their dietary choices. Consequently, the available data does not permit conclusions to be drawn about the scale or nature of consultations concerning dairy products. It is recommended that subsequent studies directly evaluate the frequency with which such consultations take place, the subjects of these consultations, and the extent to which they may influence patients’ dietary choices. This would facilitate an assessment of the actual influence of doctors in shaping the purchasing behaviour of dairy product consumers. Finally, owing to the exploratory nature of the principal component analysis, the identified three-factor structure should be considered preliminary and requires further verification.

Further research should focus on an in-depth analysis of the effectiveness of individual communication channels, particularly comparing interpersonal sources, such as doctors’ or family recommendations, with rapidly evolving digital channels. This would make it possible to determine which communication channels are most effective in reaching specific consumer groups and the extent to which the dissemination of nutritional information can contribute to changes in nutritional attitudes, intentions, and behaviours. Another interesting area for exploration would be to study the level of trust in various information sources (e.g., experts, scientific institutions, influencers and manufacturers), as well as identifying the factors that determine the responses to educational content in different age and social groups. Furthermore, future research should directly include consumers representing various demographic groups and be based on larger and more diverse samples to verify the extent to which barriers identified from the physicians’ perspective correspond to the actual attitudes, motivations, and purchasing and consumption behaviours of consumers.

## Figures and Tables

**Figure 1:**
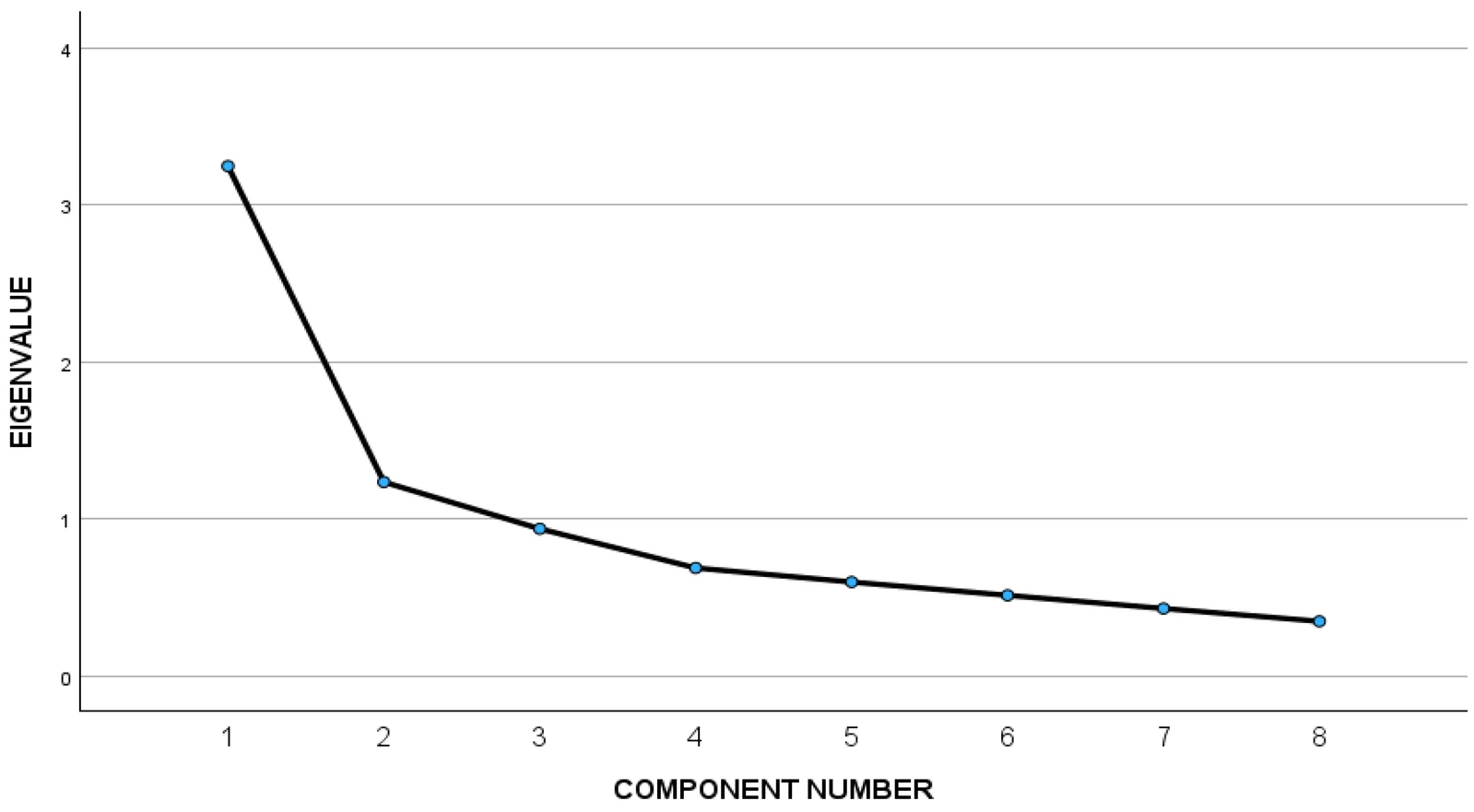
Scree plot. Source: own research results.

**Figure 2:**
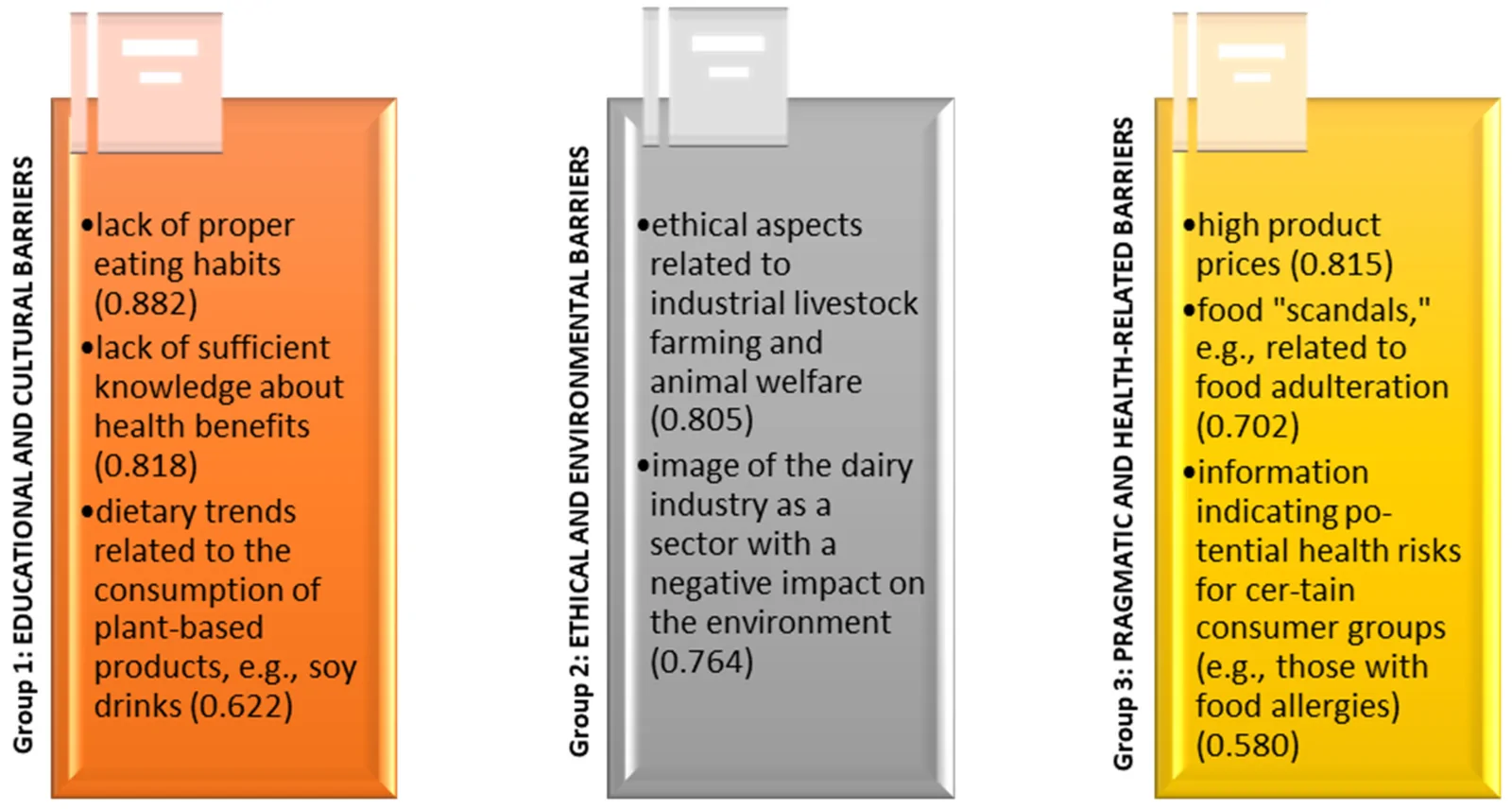
Principal components of barriers to dairy product consumption identified from physicians’ assessments. Source: own study.

**Table 1:** Factors negatively affecting the consumption of dairy products.

No.	Variables	% of Respondents					Me	M	SD	Rank
		1	2	3	4	5				
1	lack of proper eating habits	2.0	4.5	17.9	35.3	40.3	4.0	4.07	0.969	1
2	insufficient knowledge about health benefits	2.5	4.0	20.4	41.8	31.3	4.00	3.96	0.95	2
3	high product prices	1.5	12.9	32.8	36.8	15.9	4.00	3.53	0.959	7
4	image of dairy farming as a sector with a negative impact on the natural environment	3.0	14.9	29.9	35.3	16.9	4.00	3.48	1.035	8
5	industrial livestock farming	3.5	10.4	32.3	30.8	22.9	4.00	3.59	1.06	6
6	information indicating potential health risks for certain consumer groups (e.g., those with food allergies)	2.0	8.5	19.4	44.3	25.9	4.00	3.84	0.974	4
7	food scandals, e.g., related to food adulteration	2.0	10.4	17.4	38.3	31.8	4.00	3.88	1.039	3
8	dietary trends related to the consumption of plant-based products, e.g., soy drinks	3.5	10.0	21.9	38.3	26.4	4.00	3.74	1.064	5

Note: Me—median; M—arithmetic mean; SD—standard deviation; Rating scale: 1—very little impact, 2—little impact, 3—moderate impact, 4—strong impact, 5—very strong impact. Source: own research results.

**Table 2:** Principal components identified on the basis of Cattell’s scree plot criterion and the explained variance for the total sample.

Principal Components	Eigenvalue	Cumulative Eigenvalue	Variance Explained (%)	Cumulative Variance Explained
1	3.247	3.247	40.584	40.584
2	1.235	4.482	15.442	56.026
3	0.938	5.420	11.720	67.746

Note: “Total”—the Kaiser–Meyer–Olkin (KMO) measure of sample adequacy is 0.786, which is greater than 0.7. Bartlett’s sphericity test is significant (the variables are statistically significantly correlated); chi2 = 417.306; and *p* < 0.001. Source: own study.

**Table 3:** Physicians’ evaluation of sources of information and knowledge influencing the opinions and attitudes of consumers of dairy products.

No.	Variables	% of Respondents					Me	M	SD	Rank
		1	2	3	4	5				
1	doctors	2.0	3.0	27.4	36.3	31.3	4.0	3.92	0.940	2
2	scientists	1.5	10.9	27.4	31.8	28.4	4.00	3.75	1.035	5
3	social organizations	3.0	18.4	35.8	33.8	9.0	3.00	3.27	0.964	12
4	celebrities and opinion leaders	9.0	12.4	21.4	37.8	19.4	4.00	3.46	1.196	10
5	school	3.5	7.5	28.4	37.3	23.4	4.00	3.70	1.021	8
6	traditional media (radio, television, press)	2.5	10.9	26.9	37.8	21.9	4.00	3.66	1.018	9
7	the Internet	2.5	4.5	17.4	35.8	39.8	4.00	4.06	0.988	1
8	social media (e.g., Facebook, TikTok, Instagram)	6.0	4.5	21.9	32.3	35.3	4.00	3.87	1.130	3
9	family recommendations	3.0	5.5	19.4	48.8	23.4	4.00	3.84	0.946	4
10	recommendations from friends	3.5	8.5	19.4	48.8	19.9	4.00	3.73	0.989	6
11	social campaigns	1.5	8.5	27.9	41.8	20.4	4.00	3.71	0.936	7
12	recommendations from sales staff	6.5	13.4	40.3	33.8	6.0	3.00	3.19	0.968	13
13	promotional activities of producers, e.g., participation in trade fairs, sponsorship	3.0	12.4	35.3	38.3	10.9	3.00	3.42	0.946	11

Note: Me—median; M—arithmetic mean; SD—standard deviation; Rating scale: 1—very little impact, 2—little impact, 3—moderate impact, 4—strong impact, 5—very strong impact. Source: own research results.

**Table 4:** Physicians’ assessment of activities that could increase consumer interest in dairy products.

No.	Variables	% of Respondents					Me	M	SD	Rank
		1	2	3	4	5				
1	dissemination of expert opinions (scientists, doctors)	3.5	3.5	14.9	37.8	40.3	4.00	4.08	1.002	1
2	social campaigns raising awareness among children and parents	3.0	3.0	17.9	35.8	40.3	4.00	4.07	0.985	2
3	publicising the recommendations of scientific institutions regarding the benefits of consuming dairy products	4.0	3.0	15.4	37.3	40.3	4.0	4.07	1.017	2
4	awareness campaigns on quality certification labels displayed on packaging	2.5	6.0	17.4	38.3	35.8	4.0	3.99	1.000	3
5	creating a positive image of the dairy industry	3.5	6.5	22.4	36.3	31.3	4.0	3.86	1.046	4
6	highlighting environmental aspects on dairy product labels (e.g., low-emission production lines, high animal welfare, climate protection, etc.)	5.0	9.0	19.4	32.3	34.3	4.0	3.82	1.148	5
7	campaigns promoting dairy consumption with the participation of influential and renowned authors of nutrition programs and guides	6.5	8.0	24.4	33.8	27.4	4.0	3.68	1.149	5

Note: Me—median; M—arithmetic mean; SD—standard deviation. Rating scale: 1—to a very small extent, 2—to a small extent, 3—to a moderate extent, 4—to a large extent, 5—to a very large extent. Source: own research results.

## Data Availability

The data presented in this study are available on request from the corresponding author due to ethical restrictions and participant confidentiality, the data cannot be made publicly available.

## Appendix A

**Table A1:** Rotated Component Loading Matrix After Equamax Rotation.

No.	Variables	Components		
		1	2	3
1	lack of proper eating habits	0.882	0.012	0.137
2	insufficient knowledge about health benefits	0.818	0.146	0.172
3	dietary trends related to the consumption of plant-based products, e.g., soy drinks	0.622	0.359	0.22
4	industrial livestock farming	0.045	0.805	0.18
5	image of dairy farming as a sector with a negative impact on the natural environment	0.221	0.764	0.012
6	high product prices	0.221	−0.148	0.815
7	food scandals, e.g., related to food adulteration	0.112	0.419	0.702
8	information indicating potential health risks for certain consumer groups (e.g., those with food allergies)	0.179	0.49	0.58

Source: own research results.
